# Unpacking the association between chewing capacity and gallstone prevalence: a cross-sectional analysis of NHANES data

**DOI:** 10.1097/JS9.0000000000004586

**Published:** 2025-12-16

**Authors:** Yan Jiao, Ya-Hui Liu, Xiao-Feng Sun, Shu-Yu Zhou, Bo Liu

**Affiliations:** a Department of Hepatobiliary and Pancreatic Surgery, General Surgery Center, The First Hospital of Jilin University, Changchun, Jilin, China; b Department of Cadre’s Wards Ultrasound Diagnostics, Ultrasound Diagnostic Center, The First Hospital of Jilin University, Changchun, Jilin, China; c School of Pharmacy, Beihua University, Jilin, China

**Keywords:** chewing capacity, epidemiology, functional tooth unit, gallstone

## Abstract

**Background:**

Gallstone disease has been identified as a prevalent clinical entity in emergency general surgery, and tooth loss is considered a potential risk factor for gallstone disease; the relationship between chewing capacity and gallstone formation remains unexplored. As traditional tooth counts inadequately assess chewing capacity, we employ functional tooth units (FTUs), a validated biomechanical metric, to investigate the chewing capacity-gallstone association through multivariate epidemiological analysis.

**Method:**

This study used data from the National Health and Nutrition Examination Survey 2017–2020. Chewing capacity was evaluated based on the number of FTU, which were defined as pairs of opposing natural and artificial teeth. Gallstone status was determined based on abdominal sonography. Logistic regression analyses, subgroup analyses, and restricted cubic spline regression were conducted to assess the association between impaired chewing capacity (ICC) and gallstones.

**Results:**

An association between FTU and the risk of gallstones was detected (odds ratio, OR = 0.90, 95% confidence interval [CI]: 0.85–0.96), suggesting protective roles of more FTUs. Significant increase in the risk of gallstones in ICC was observed (OR = 1.81, 95% CI: 1.27–2.57). Subgroup analyses demonstrated consistent results in all subgroups, except for individuals aged >65, male, white, education (Grades 0–12, High school or equivalent), drinking, Normal weight, Overweight, and diabetes. Restricted cubic spline regression analysis demonstrated that the risk of gallstones increased with decreasing FTUs, particularly for FTU ≤6.

**Conclusions:**

This population-based study revealed a significant link between the ICC and a higher prevalence of gallstones, which suggests that maintaining functional teeth may be a protective factor. Prospective studies are needed to elucidate the causal mechanisms and explore potential intervention strategies.

## Introduction

Gallstone disease has long been one of the most common diseases of the digestive system, and the incidence of gallstones ranges from 10% to 20% in adults^[1,2]^. Gallstones frequently lead to acute cholecystitis, biliary duct obstruction, and biliary pancreatitis, which represent potentially life-threatening conditions that necessitate urgent clinical intervention in the emergency department^[3,4]^. In recent years, the incidence rate of gallstones has been increasing with changes in people’s lifestyle and diet, which leads to socioeconomic costs that have also been rising^[5]^.

**HIGHLIGHTS**

- Functional tooth unit, a more reliable indicator, was used to determine chewing capacity.
- Summarized the demographic and lifestyle factors related to gallstones.
- Moderate declines in chewing capacity may significantly stimulate gallstone formation.

Although the risk factors of gallstone formation have been widely studied, new research showed that chewing capacity plays a key role in the process of digestion and metabolism, potentially affecting the formation of gallstones^[6–8]^. Moreover, some research has reported that the prevalence of gallstones is higher in individuals with impaired chewing capacity (ICC)^[9,10]^. However, those studies have determined chewing capacity by roughly counting the number of teeth lost or by a self-reported approach^[11]^. A more reliable indicator has been proposed – functional tooth unit (FTU), which measures posterior occlusal support based on opposing premolar/molar pairs^[12,13]^.

Although there is a potential relationship between chewing capacity and gallstones, previous epidemiological studies mainly focused on the traditional metabolic risk factors, and limited consideration of chewing capacity as a contributing factor^[14,15]^. In view of the complex interaction between chewing capacity, diet, and metabolic health, it is crucial to explore whether chewing capacity is an independent risk factor for gallstone formation. We hypothesize that chewing capacity, as evaluated by the number of FTU, was independently related to the prevalence of gallstones, and that this association remains significant after adjusting for demographic, lifestyle, and metabolic confounders. Additionally, we explore the potential non-linear association between FTUs and the risk of gallstones, and identify the potential threshold effects of chewing capacity on gallstone formation.

## Method

### Study design and participants

**Figure 1.**
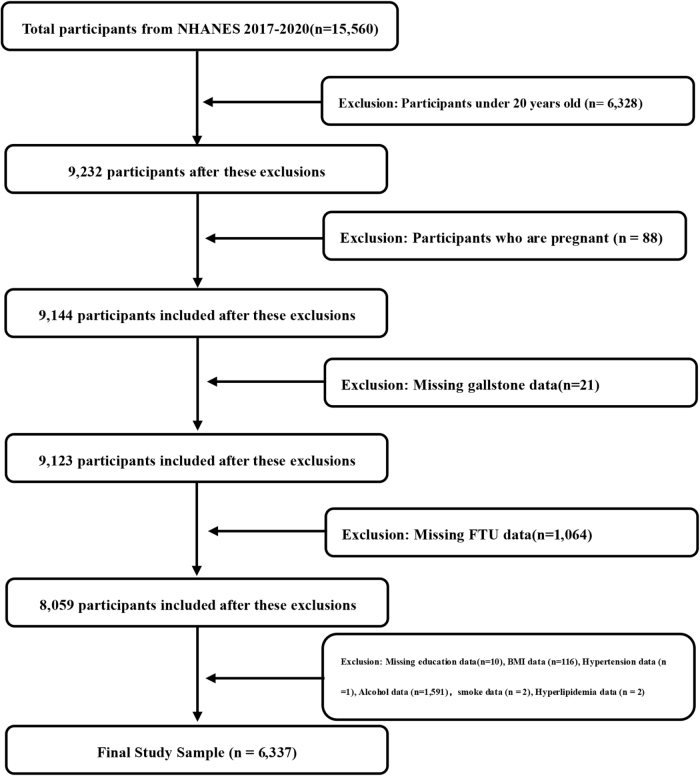
Flowchart of participant selection and composition. BMI, body mass index; NHANES, National Health and Nutrition Examination Survey; FTU, functional tooth unit.

This study utilized data from the National Health and Nutrition Examination Survey (NHANES) 2017–2020. All data and guidelines are publicly available through https://www.cdc.gov/nchs/nhanes/index.htm. Participants were excluded if they were under 20 years of age (*n* = 6,328), pregnant (*n* = 88), or had missing diagnostic data of gallstones (*n* = 21). Additional exclusions were applied for missing FTU data (*n* = 1064), missing information on education (*n* = 10), body mass index (BMI) (*n* = 116), hypertension status (*n* = 1), smoke (*n* = 2), hyperlipidemia (*n* = 2) and alcohol consumption (*n* = 1591), leading to a final analytical sample of 6337 participants. The process of study design and participant selection is shown in Figure 1. The work has been reported in line with the STROCSS criteria and the TITAN criteria^[16,17]^.

### Assessment of chewing capacity

Chewing capacity was evaluated based on the number of FTUs. One FTU was two opposing teeth in these posterior teeth, which included premolars and molars, and the total FTU score ranged from 0 to 8. Participants were classified into two groups according to prior literature, one with FTU ≥5 defined as adequate chewing capacity (ACC), and the other for ICC with FTU ≤4, as reported in previous studies^[18,19]^.

### Gallstone diagnosis

Gallstone status was determined based on abdominal sonography performed during NHANES examinations. Participants were categorized as gallstone-positive if gallstones were detected via ultrasound or if they had a history of cholecystectomy. Participants without gallstones detected on ultrasound and without a history of gallbladder removal were classified as gallstone-negative.

### Covariates

To adjust for potential confounding factors, several covariates were included in the analysis. Demographic variables included age, gender, race, and educational attainment. Lifestyle factors included alcohol and cigarette consumption, categorized as either non-drinker or drinker, non-smoker or smoker, and weight status, categorized based on BMI into normal weight (<25), overweight (25–29.9), and obese (≥30). Health conditions included hypertension, hyperlipidemia, and diabetes, both categorized as present or absent based on self-reported physician diagnoses.

### Statistical analysis

All statistical analyses were carried out using R software. Continuous variables were presented as means with 95% confidence intervals (CIs) and compared using *t*-tests or Mann–Whitney *U* tests, while categorical variables were presented as counts and percentages and compared using chi-square tests. The association between chewing capacity and gallstone prevalence was assessed using logistic regression models. The crude model included only the FTU classification. Model 1 adjusted for age, gender, race, and education. Model 2 further adjusted for drinking, smoking, and weight status. Model 3 included additional adjustments for hypertension, hyperlipidemia, and diabetes to account for potential metabolic influences on gallstone formation. To explore potential non-linear associations between FTUs and gallstone prevalence, restricted cubic spline regression was conducted, with statistical significance for non-linearity assessed using likelihood ratio tests.

## Results

### General characteristics of study population participants

Table 1 demonstrates the general characteristics of these participants. Among gallstone patients, the average age was 57.82, with females accounting for 51.84%, and a predominance of White (63.42%). Compared to non-gallstone controls, self-reported gallstone patients were more likely to be older, had a higher BMI, predominantly female, unhealthy lifestyles (e.g. smoking), but less likely to drink. About comorbidities, gallstone patients had a higher prevalence of diabetes, hypertension, and hyperlipidemia. Regarding chewing capacity, gallstone patients had a lower FTUs (4.50), and a higher proportion of ICC (44.91%).

**Table 1:** General characteristics of study population participants grouped by gallstone in the National Health and Nutrition Examination Survey, 2017–2020.

Variable	Total	Gallstone		*P*-value
		No	Yes	
Age	48.52 (47.34–49.70)	47.37 (46.15–48.59)	57.82 (56.39–59.26)	<0.0001
Age group				<0.0001
≤65	4772 (78.10)	4354 (80.24)	418 (60.72)	
>65	1565 (21.90)	1292 (19.76)	273 (39.28)	
Gender				<0.0001
Male	3069 (48.52)	2869 (51.07)	200 (27.86)	
Female	3268 (51.48)	2777 (48.93)	491 (72.14)	
Race				0.05
White	2309 (63.42)	1999 (62.67)	310 (69.50)	
Black	1782 (11.39)	1633 (11.82)	149 (7.89)	
Mexican	687 (7.84)	608 (7.92)	79 (7.18)	
Other	1559 (17.35)	1406 (17.59)	153 (15.43)	
Education				0.01
Grades 0–12	1004 (9.22)	900 (9.40)	104 (7.79)	
High school or equivalent	1504 (26.59)	1329 (25.67)	175 (34.06)	
College or above	3829 (64.18)	3417 (64.93)	412 (58.15)	
Drinking				0.001
No	5306 (78.83)	4699 (77.92)	607 (86.27)	
Yes	1031 (21.17)	947 (22.08)	84 (13.73)	
Smoking				0.01
No	3659 (58.37)	3295 (59.07)	364 (52.70)	
Yes	2678 (41.63)	2351 (40.93)	327 (47.30)	
BMI	29.74 (29.37–30.11)	29.36 (29.04–29.68)	32.80 (31.96–33.64)	<0.0001
Weight status				<0.0001
Normal weight	1565 (26.38)	1486 (28.00)	79 (13.19)	
Overweight	1949 (31.00)	1766 (31.19)	183 (29.40)	
Obese	2823 (42.62)	2396 (40.80)	429 (57.41)	
Hypertension				<0.0001
No	3383 (60.82)	3126 (63.18)	257 (41.61)	
Yes	2954 (39.18)	2520 (36.82)	434 (58.39)	
Diabetes				<0.0001
No	5105 (85.20)	4639 (86.58)	466 (73.95)	
Yes	1232 (14.80)	1007 (13.42)	225 (26.05)	
Hyperlipidemia				<0.001
No	2117 (34.52)	1954 (35.97)	163 (22.76)	
Yes	4220 (65.48)	3692 (64.03)	528 (77.24)	
FTUs	5.72 (5.53–5.91)	5.87 (5.68–6.06)	4.50 (4.16–4.84)	<0.0001
FTU				<0.0001
ACC	4054 (73.37)	3712 (75.62)	342 (55.09)	
ICC	2283 (26.63)	1934 (24.38)	349 (44.91)	

ACC, adequate chewing capacity; BMI, body mass index; FTU, functional tooth unit; ICC, impaired chewing capacity.

### Association between chewing capacity and gallstones

Table 2 demonstrates the logistic regression analysis between chewing capacity and gallstones. In the crude model, FTU was significantly associated with decreased odds of gallstone (95% CI = 0.85). When adjusting demographic confounders in Model 1, the above correlation still exists (95% CI = 0.88). Model 2 adjusted for living habits, FTU was still significantly associated with reduced odds of gallstone (95% CI = 0.88). Model 3 adjusted for health conditions, there was still a significant association between FTU and reduced risk of gallstones (95% CI = 0.90).

**Table 2:** The association between the chewing capacity and gallstones.

Variable	Crude model		Model 1		Model 2		Model 3	
	95% CI	*P*	95% CI	*P*	95% CI	*P*	95% CI	*P*
FTU	0.85 (0.82–0.89)	<0.0001	0.88 (0.83–0.93)	<0.001	0.88 (0.84–0.93)	<0.001	0.90 (0.85–0.96)	0.003
ICC	2.53 (1.94–3.30)	<0.0001	2.16 (1.50–3.10)	<0.001	1.91 (1.35–2.71)	0.001	1.81 (1.27–2.57)	0.003
ACC	ref		ref		ref		ref	

Crude model: Unadjusted. Model 1: Adjusted for age, gender, race, education. Model 2: Adjusted for age, gender, race, education, drinking, weight status, smoking. Model 3: Adjusted for age, gender, race, education, drinking, weight status, hypertension, diabetes, hyperlipidemia.

FTU was divided into two groups for further analyses, including ICC and ACC. Consistent with the above correlation, a significant increase in the risk of gallstones in ICC was observed compared with ACC in all Models (Crude model: 95% CI = 2.53; Model 1:95% CI = 2.16; Model 2: 95% CI = 1.91; Model 3:95% CI = 1.81).

### Subgroup analyses by potential effect modifiers

**Figure 2.**
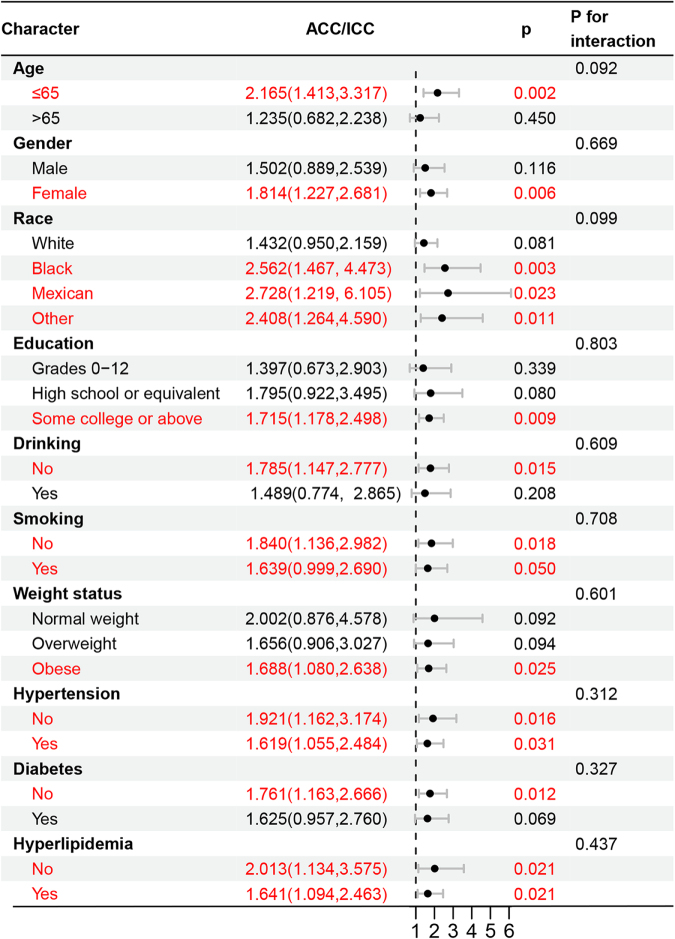
Subgroup analyses by possible effect modifiers for the relationship between chewing capacity and gallstones.

Subgroup analyses were performed to evaluate the potential modifying effects of age, gender, BMI, smoking, drinking, and disease history on the association between chewing capacity and gallstone risk. A significant negative association was consistently observed in the following subgroups: individuals aged ≤65 years, females, those of Black, Mexican, or other racial backgrounds, individuals with some college or above, non-drinkers, both smokers and non-smokers, those with obesity, participants with or without hypertension, those with or without hyperlipidemia, and non-diabetic individuals (Fig. 2).

### Non-linear relationship between FTU and gallstone risk

**Figure 3.**
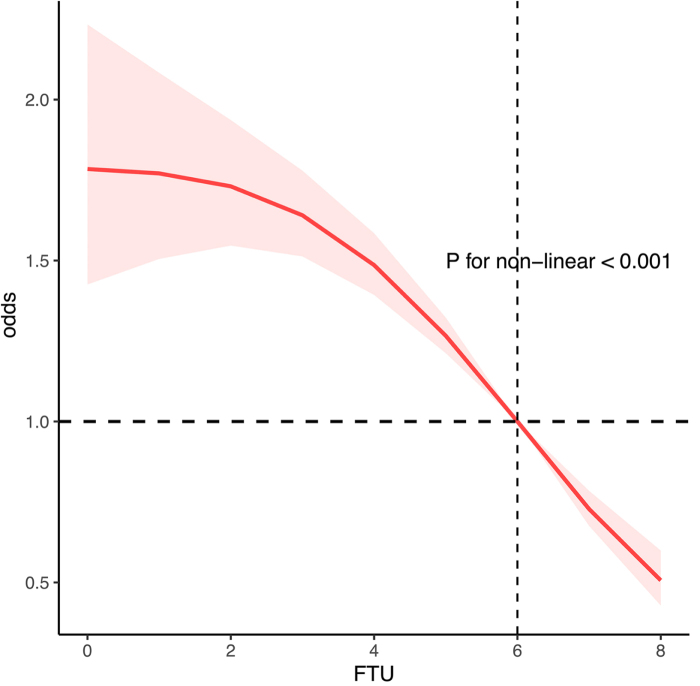
Association between functional tooth unit (FTU) and gallstones.

Restricted cubic spline regression analysis demonstrated a significant non-linear association between FTU and gallstone prevalence (*P* for nonlinearity <0.001). The risk of gallstones increased with decreasing FTUs, particularly FTU ≤6 (Fig. 3).

## Discussion

Consistent with previous studies, gallstones are also influenced by various demographic and lifestyle factors^[20,21]^. For example, age plays a big role because gallstones are more likely to develop with age^[22]^. Gender is also a contributing factor, and women are more likely to suffer from gallstones than men^[23]^. BMI also plays a role, as obesity could increase the risk of gallstones formation^[24]^. Other modifiable factors, such as smoking, rather than drinking, were associated with increased risks of gallstones^[25]^. Furthermore, hypertension, diabetes, and hyperlipidemia, also play a major role in gallstone formation^[26,27]^.

Previous investigations have indicated a correlation between the number of missing teeth and the prevalence of gallstones^[10]^. However, the number of teeth can not reflect the chewing capacity properly. The findings of this study provide novel epidemiological evidence supporting an independent association between FTU and gallstone risk, suggesting that individuals with ICC exhibited a higher susceptibility to gallstones.

A growing body of research now offers insights into how diminished chewing capacity may biologically contribute to gallstone formation. Impaired mastication has been shown to disrupt the oral–gut microbial axis, particularly enriching bile-acid–modifying taxa such as Desulfovibrionales and Proteobacteria, which increase secondary bile acids and promote more hydrophobic, lithogenic bile profiles^[28,29]^. These microbial alterations can influence hepatic FXR signaling and downregulate CYP7A1, ultimately disrupting bile acid synthesis and enhancing biliary cholesterol secretion^[28,30]^. In parallel, dysbiosis-related metabolites – including hydrogen sulfide and lipopolysaccharides – may trigger systemic inflammation and metabolic endotoxemia, further disturbing cholesterol homeostasis and increasing the likelihood of biliary cholesterol supersaturation^[31,32]^.

In addition to these metabolic effects, impaired chewing may also influence biliary physiology through changes in gastrointestinal motility. Altered microbial composition and bile acid profiles have been associated with delayed intestinal transit and reduced gallbladder contractility, both of which prolong bile residence time and raise levels of deoxycholic acid – an established driver of cholesterol crystallization^[33,34]^. Taken together, these findings suggest that microbial dysregulation, bile acid disturbances, and impaired gallbladder motility may jointly provide a plausible mechanistic basis for the observed association between reduced chewing capacity and gallstone development.

Furthermore, the significant non-linear correlation between FTUs and gallstone risk, showing that even moderate declines in chewing capacity may contribute to gallstone development. From a public health perspective, this finding highlights chewing capacity as a potentially modifiable target for gallstone prevention. More concrete intervention strategies may therefore be considered, such as integrating routine oral health assessments into metabolic disease management programs, implementing early FTU screening for individuals with recognized gallstone risk factors, and incorporating dental rehabilitation or chewing-training interventions for patients with FTU ≤6. In addition, nutritional counseling that emphasizes fat-controlled and fiber-rich dietary patterns for individuals with impaired chewing capacity may help mitigate dietary pathways contributing to gallstone formation.

Importantly, existing clinical guidelines – such as the EASL Clinical Practice Guidelines on the Prevention, Diagnosis and Treatment of Gallstones – primarily emphasize weight management, dietary modification, control of metabolic risk factors, and appropriate management of biliary symptoms, while not addressing chewing function or oral-health–related factors in gallstone prevention^[35]^. However, these guidelines do not mention chewing capacity, FTU evaluation, or oral health–related interventions as potential preventive strategies. This highlights the novelty of our findings and suggests that incorporating chewing function into current prevention frameworks may complement existing guideline recommendations and broaden preventive approaches for gallstone disease. Clinically, dentists and physicians should be aware of the potential systemic consequences of tooth loss and impaired chewing.

There are some limitations in our research. For example, the cross-sectional study design prevents the exploration of causal associations. Some analyses show no significant association in populations, which may be related to small sample sizes or incompletely controlled confounding factors. Due to limitations in NHANES data, the mobility of teeth, periodontitis, status of masticatory muscles, and dental caries were not taken into account, which may have led to an inaccurate representation of chewing capacity. Furthermore, dietary structure is not considered, potentially missing confounding pathways. Prospective studies are needed to elucidate this relationship further.

## Conclusion

This population-based study revealed a significant link between the ICC and a higher prevalence of gallstones, which suggests that maintaining functional teeth may be a protective factor. Prospective studies are needed to elucidate the causal mechanisms and explore potential intervention strategies.

## Data Availability

The datasets generated during the current study are available from the corresponding author on reasonable request. The data that support the findings of this study are openly available in NHANES at https://www.cdc.gov/nchs/nhanes/index.htm.
